# A weight-of-evidence approach to assess chemicals: case study on the assessment of persistence of 4,6-substituted phenolic benzotriazoles in the environment

**DOI:** 10.1186/s12302-016-0072-y

**Published:** 2016-02-11

**Authors:** Marc Brandt, Eva Becker, Ulrich Jöhncke, Daniel Sättler, Christoph Schulte

**Affiliations:** German Environment Agency, Section IV 2.3 “Chemicals”, Umweltbundesamt (UBA), Wörlitzer Platz 1, 06844 Dessau-Roßlau, Germany

**Keywords:** Risk assessment, Weight-of-evidence approach, Persistence, Phenolic benzotriazoles, REACH, SVHC, Read-across, Monitoring studies, QSAR

## Abstract

**Background:**

One important purpose of the European REACH Regulation (EC No. 1907/2006) is to promote the use of alternative methods for assessment of hazards of substances in order to avoid animal testing. Experience with environmental hazard assessment under REACH shows that efficient alternative methods are needed in order to assess chemicals when standard test data are missing. One such assessment method is the weight-of-evidence (WoE) approach. In this study, the WoE approach was used to assess the persistence of certain phenolic benzotriazoles, a group of substances including also such of very high concern (SVHC).

**Results:**

For phenolic benzotriazoles, assessment of the environmental persistence is challenging as standard information, i.e. simulation tests on biodegradation are not available. Thus, the WoE approach was used: overall information resulting from many sources was considered, and individual uncertainties of each source analysed separately. In a second step, all information was aggregated giving an overall picture of persistence to assess the degradability of the phenolic benzotriazoles under consideration although the reliability of individual sources was incomplete.

**Conclusions:**

Overall, the evidence suggesting that phenolic benzotriazoles are very persistent in the environment is unambiguous. This was demonstrated by a WoE approach considering the prerequisites of REACH by combining several limited information sources. The combination enabled a clear overall assessment which can be reliably used for SVHC identification. Finally, it is recommended to include WoE approaches as an important tool in future environmental risk assessments.

## Background

In the European Union, chemicals are subject to the chemicals legislation REACH (EC 1907/2006) [1]. Manufacturers and importers have to register substances to the European Chemicals Agency (ECHA) and provide the risk assessment as required by REACH. The authorities are responsible to evaluate certain selected substances and—if necessary—to propose and enforce additional regulatory actions like authorisation or restriction of chemicals and their uses. Of special interest are substances of very high concern (SVHC). With regard to the environment, SVHC mostly are substances that are identified as persistent, bioaccumulative and toxic (PBT substances) and substances that are very persistent and very bioaccumulative (vPvB substances). SVHC are identified under REACH in a formal process. A Member State or the ECHA needs to demonstrate that the criteria laid out in REACH Annex XIII are fulfilled (see Table 1). A main policy goal of REACH is “to ensure a high level of protection of human health and the environment, including the promotion of alternative methods for assessment of hazards of substances” (REACH Article 1).

**Table 1:** Criteria for assessment of PBT and vPvB properties according to REACH Annex XIII, number 1

	PBT	vPvB
Persistence		
Screening criterion	Not readily biodegradable	
	Degradation half-lives (days)	
Water, marine	>60	>60
Fresh water	>40	>60
Marine sediment	>180	>180
Fresh water sediment	>120	>180
Soil	>120	>180
Bioaccumulation		
Screening criterion	Log *K*_ow_ >4.5	
BCF	>2000	>5000
Toxicity	NOEC <0.01 mg/l	–
	CMR	
	Endocrine active	

This article focuses on one specific alternative method for the assessment of hazards of substances, the weight-of-evidence approach (WoE approach). In addition, some other alternative methods for prediction of chemical properties like read-across assessment or use of in silico methods are briefly described.

Two reasons support the use of alternative assessment methods: alternative methods for hazard assessment are necessary, as animal testing should be avoided if possible. On the other hand, there is the problem that for specific situations reliable information needed for assessing the hazard potential of a substance is missed when only the established standard assessment schemes, which consist mostly of laboratory tests, are used. The latter leads to misjudgment of substances, which need to be identified as SVHC. To illustrate this point, a small number exercise can be employed: there is an obvious mismatch between the number of substances registered under REACH and the number of substances identified as PBT/vPvB. Currently, 9032 substances (as of the 31.12.2015) are registered under REACH. However, there are only 22 substances (as of 3.12.2015) identified as PBT and/or vPvB substances so far and not all of them have been registered. If all of these would have been registered, that would amount to 0.2 % of the registered substances. In a study by Strempel et al. [2], 94,483 substances of the European Inventory of Existing Commercial chemical Substances (EINECS register) were screened for PBT and vPvB properties. Based on their results, the authors estimate that approximately 3 % of all the substances in the register, i.e. 2930 substances, might be PBT/vPvB substances. In comparison to the 22 PBT/vPvB substances currently identified, this is a mismatch that can only partially be explained by the fact that the REACH registration has been in force for merely 9 years.

In 2010, the review of the setup of procedures to identify SVHC concluded that these cannot ensure the policy goal of a sufficiently high level of protection. Therefore, in 2011 Annex XIII was amended in order to enable identification of SVHC according to the state of environmental science [3]. The amended Annex XIII strengthens the assessment by allowing additional endpoints indicating PBT properties (e.g. biomagnification) and different assessment strategies like the WoE approach.

Up to now, there is not much experience in employing the WoE approach under REACH although this approach has been used in the past 60 years, especially in medicine, but also in toxicology (see for example [4]) and at least since the 1990s in ecological risk assessment (see for example [5–7]). One reason for its elusiveness might be its ambiguous definition and use. Linkov et al. [8] analysed weight-of-evidence evaluations and came up with a classification system based on the amount of use of qualitative and quantitative methods. In a review of the WoE approach by Weed [9], at least three different interpretations were found in 92 selected WoE studies conducted between 1994 and 2004: the term WoE approach is either meant simply as a metaphor, as a methodology (but Weed also defines subcategories of this interpretation) or as a theory. In the REACH regulation, the WoE approach is defined as follows: “There may be sufficient weight of evidence from several independent sources of information leading to the assumption/conclusion that a substance has or has not a particular dangerous property, while the information from each single source alone is regarded insufficient to support this notion”. With regard to the categorisation proposed by Weed, this means that the WoE approach in REACH is what he calls a “summary narrative”: after presenting all available information, there is another step where all pieces are assessed as individual lines of evidence that culminate in a conclusion. The individual lines might be weighted but this has to be done in a qualitative way.

There are of course other ways for performing a weight-of-evidence approach, especially such where the weighing of information is done quantitatively (e.g. [10]). In addition, recently the hypothesis-based WoE approach proposed by Rhomberg et al. [11] has found strong support in the literature (see for example [12]).

**Fig. 1:**
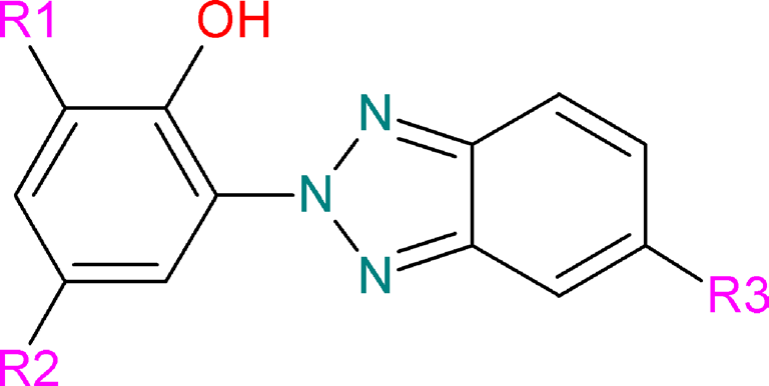
Generalized structures of the phenolic benzotriazoles; R1: H, alkyl, alkylaryl; R2: alkyl, alkylaryl and R3: H, Cl

Nevertheless, in this article we discuss the use of the WoE approach according to the “summary narrative” of Weed as we believe that this approach is best suited for a flexible WoE application in regulatory proposals under REACH according to its definition. Implicitly, this constitutes a hypothesis-based WoE where the hypothesis is the existence of a certain hazard profile. Given the diverse nature of information employed for our case example, quantitative weighting would be hardly possible in an objective way. The case example presented here is the part of the regulatory work on the four phenolic benzotriazoles: UV-320, UV-327, UV-328 and UV-350 (see Fig. 1; Table 2) that were recently assessed by the German Environment Agency and then submitted as SVHC proposals by Germany. The according dossiers are available online at the homepage of ECHA [13–16]. They contain these assessments of an overall environmental hazard assessment on PBT-related properties and additional data as well as studies not of relevance for the present discussion.

**Table 2:** Overview of phenolic benzotriazoles discussed in this article

Name	CAS no.	EC no.	R1	R2	R3
UV-320	3846-71-7	223-346-6	*Tert*-butyl	*Tert*-butyl	H
UV-327	3864-99-1	223-383-8	*Tert*-butyl	*Tert*-butyl	Cl
UV-328	25973-55-1	247-384-8	*Tert*-pentyl	*Tert*-pentyl	H
UV-350	36437-37-3	253-037-1	*Sec*-butyl	*Tert*-butyl	H

This case example is illustrative as it highlights potential strategies to employ when other established hazard assessment strategies fail. The substances in question were suspected to be SVHC, but they could not be formally identified as PBT/vPvB substances based on the numerical criteria of REACH Annex XIII, because some of the required experimental data are not available. In this case, degradation half-lives were missing which is probably due to the experimental complexity necessary for their accurate determination. As there were no adequate half-lives from other studies, e.g. field or monitoring studies available, the WoE approach was used to assess the persistence. In the terminology of the hypothesis-based approach, the working hypothesis was that the substances in question were assumed to be very persistent in the environment. In the definition used in REACH, this means that their degradation half-lives in the different compartments water, soil and/or sediment would be above 180 days when tested according to standard simulation tests on degradation.

In this article, we present the methodology employed, a brief description of the experiences made during expert discussions at EU level and the still open questions remaining.

## Results and discussion

### Definition of phenolic benzotriazoles

In this study, phenolic benzotriazoles are defined as 2-(2H-benzotriazole-2-yl)-phenols that are substituted in position 4 and/or 6, mostly by different bulky alkyl or alkylaryl substituents. Overall, there are around two dozen different substances on the market that meet the definition used here. In this article, mainly the four phenolic benzotriazoles UV-320, UV-327, UV-328 and UV-350 are discussed (see Fig. 1; Table 2).

Phenolic benzotriazoles are used as UV stabilisers since they absorb the full spectrum of UV light. At the molecular level, UV radiation excites the phenolic benzotriazole. In this excited state, a proton from the OH group is transferred to a nitrogen atom. From this structure, a radiationless deactivation coupled with another proton transfer from the nitrogen back to the OH group will bring the molecule back to its ground state. The UV protection properties are based on this fully reversible and non-destructive process. Besides the group of benzophenones, phenolic benzotriazoles are technically the most important UV absorbers, especially for transparent plastic materials and coatings but also in certain personal care products. The different substitution patterns in ortho and para position to the hydroxyl group of the phenolic ring have effects on the solubility and the distinct coloration in different transparent plastic materials [17].

Substances of this chemical group have been repeatedly addressed as emerging contaminants. In Japan, two substances of this group are regulated. Manufacture, import and use of UV-320 (CAS 3846-71-7; see Table 1) are restricted due to PBT properties according to the Japanese definition [18]. For UV-327 (CAS 3864-99-1; see Table 1), quantities manufactured and imported have to be notified and further testing is required due to its structural similarity to UV-320 and its thereof expected high potential for bioaccumulation. UV-320, UV-328 (CAS 25973-55-1; see Table 1) and UV-329 (CAS 3147-75-9; R1, R3:H; R2: 1,1,3,3-tetramethylbutyl) are listed under the Convention for the Protection of the marine Environment of the North-East Atlantic (OSPAR Convention) as substances of possible concern, i.e. substances which warrant further work by OSPAR [19]. Furthermore, in several reports published by the US Agencies concerns are raised regarding certain phenolic benzotriazoles [20, 21]. In 2010, the British Environment Agency published results of a report applying Quantitative Structure–Activity Relationship (QSAR) methods and indicating that at least some substances of this group might have properties of high concern for the environment [22]. Finally, there is also a large collection of numerous findings of phenolic benzotriazoles in different environmental compartments and biota in many countries (see for example [23–29]).

Although phenolic benzotriazoles are of some commercial importance and have already been discussed in literature as emerging contaminants, potential persistent organic pollutants and PBT substances (see for example [30, 31]), there are still only few experimental physico-chemical data available. A brief overview of those of the four substances in question is given in Table 3. In general, phenolic benzotriazoles are solid, have a low vapour pressure and a very low water solubility, but high log *K*_OW_ and log *K*_OC_ values. They have a high bioaccumulation potential in organisms [13–16] and there is evidence of ecotoxicological effects (see for example [35]). Furthermore, ECHA’s scientific Risk Assessment Committee concluded that based on available studies UV-320 and UV-328 show specific target organ toxicity following repeated exposure [36].

**Table 3:** Overview of some experimental physico-chemical data for UV-320, UV-327, UV-328 and UV-350

	UV-320	UV-327	UV-328	UV-350
Mol. weight (g/mol)	323.4	357.9	351.5	323.4
Melting/freezing point (°C)	Not available	154–156 [32]	81.2 [33]	81–83 [32]
Boiling point (°C)	Not available	Not available	>180 [33]	Not available
Log *K*_ow_	Not available	Not available	>6.5 [33]	Not available
Water sol. (mg/l)	Not available	0.022 [34]	<0.001 at 20 °C [33]	Not available
Vapour pressure (Pa)	Not available	Not available	0.000005 at 20 °C [33]	Not available

UV-320, UV-327 and UV-328 are found in a variety of environmental compartments and in biota in many countries. As UV-350 was only seldom included in monitoring studies, no such statement can be made for it.

### Assessment strategy and individual weighing of the information given by each source

As a first step of the WoE assessment, the information given by each source is presented and individually assessed. This WoE approach is based on five different elements: screening studies on persistence and QSAR estimations are the first element. The second–fourth elements are the assessment of three independent simulation studies of which neither can be used on its own to determine the degradation half-lives of the substance since there are always deficiencies. One is a degradation test on the substances in question under laboratory conditions, one is a field study of the dissipation behaviour and one study is on the disappearance of a similar substance. The final element of the WoE approach is a case example of findings in the environment in Rhodes Island, USA, covering several decades.

As mentioned above, the overall WoE approach is that of the narrative summary meaning that the assessment is done in a purely qualitative way.

Assessments using (semi-)quantitative scoring schemes are often considered superior due to the alleged objectivity of the quantitative assessment. While such methods have certain advantages, for regulatory purposes like the one presented here our experience shows that a qualitative approach is actually better suited. A quantitative assessment would have to encompass some scoring or weighting of the available data. In order to do this, these criteria, their scores or weights and the ranges when which score has to be reached would have to be defined which introduces subjectivity when done within an assessment itself. Also, within a regulatory decision process like the SVHC identification in REACH, it would be necessary to seek agreement on the method of assessment as well as the conclusions of it. In comparison, within the narrative summary used here the chain of argumentation, deviant results and uncertainties are described directly without prior translation in scores. Thus, the focus of regulatory decision-making processes is purely on these items rather than on a meta-discussion of the employed method.

Thus ideally, method definitions discussions would have to be completed prior to individual assessments. While this also improves the overall objectivity, even with a set of defined rules there is room for subjectivity, e.g. through a certain choice of presenting studies.

Currently, under REACH such a quantitative WoE scheme is missing. Certainly, there are arguments like for example improved objectivity that speaks for the development of such a scheme once enough experience is available with handling WoE assessments. However, one major drawback of this development would be the loss of flexibility that comes with it. Under REACH, regulators have to use the data available within the system. The generation of additional data is only possible within certain boundaries in the process of substance evaluation (Title VI, Chapter 2 of REACH). This is a resource- and time-consuming procedure for all involved actors. The open definition of what constitutes a WoE cited above represents a legally intended way of dealing with this situation in a flexible manner. The setting of certain criteria, scores or ranges and enforcing quantification would lead to a loss of this flexibility.

#### Experimental tests and in silico simulations of ready biodegradability

For three of the four substances, tests on ready biodegradability are available. For UV-320 and UV-327, tests according to the OECD 301 C test protocol [Modified MITI (I) Test] are available. Both substances have no biological oxygen demand at all, i.e. there was no mineralization via mainly biological degradation within the 28 days of the test. In a test according to the OECD 301 B test protocol (Modified Sturm Test), UV-328 showed only 2–8 % degradation in 28 days meaning that the substance is not readily biodegradable [37–39].

For all four phenolic benzotriazoles, the degradability was calculated using different QSAR models as well. The results are shown in Table 4. In the REACH guidance R.11, two screening criteria are defined as prerequisites for QSAR calculations using the model EPISUITE [40]. They require that the result of the BIOWIN2 model is <0.5 as well as that of the BIOWIN3 model is <2.2 or that of the BIOWIN6 model is <0.5 as well as that of the BIOWIN3 model is <2.2. These criteria are met for UV-320, UV-327 and UV-328. UV-350 is a borderline case. However, one essential group for the degradation of the phenolic benzotriazoles, the triazole group itself, is not represented in the models. As the group is known to be difficult to degrade [41], the effect of this group in the molecule will be an inhibition of biological degradation. Hence, UV-350 is assessed as not readily biodegradable as well. Thus, the results of the QSAR simulations are in accordance with the screening tests.

**Table 4:** Results of QSAR calculations on ready biodegradation (all results are rated as reliable with restrictions)

	UV-320	UV-327	UV-328	UV-350
BIOWIN 2	0.016 (does not biodegrade fast)	0.0013 (does not biodegrade fast)	0.0108 (does not biodegrade fast)	0.1329 (does not biodegrade fast)
BIOWIN 6	0.0091 (not readily biodegradable)	0.0024 (not readily biodegradable)	0.0096 (not readily biodegradable)	0.012 (not readily biodegradable)
BIOWIN 3	1.1165 (months)	1.8338 (>1 month)	2.0546 (months)	2.2538 (weeks–months)
Overall conclusion acc. to QSAR criteria of ECHA guidance R.11^a^	Screening criterion for persistence met	Screening criterion for persistence met	Screening criterion for persistence met	Screening criterion for persistence not met, borderline case

^a^(BIOWIN2 <0.5 AND BIOWIN3 <2.2) OR (BIOWIN6 <0.5 AND BIOWIN 3 <2.2)

#### Simulation laboratory study similar to OECD 308

Wick et al. (publication accepted) investigated the biodegradation of several phenolic benzotriazoles including the four substances in question in a water–sediment study. This non-GLP study followed largely the test method OECD 308. The results of this study are shown in Table 5. Due to a contamination of the sample, a further analysis of UV-320 was not possible. Analysis of the soluble and adsorbed concentrations confirmed a high sorption affinity of the substances (see also [13]). Nevertheless, due to the use of an intensive extraction method, which was specifically designed to recover as much of the non-radioactive test substance as possible, there were practically no “non-extractable residues” (NERs). As the substance rapidly dissipates into the sediment, the DT_50_ in water can be expected to be <2 days for all substances, while DT_50_ in sediment and in the total system was far beyond 100 days. The formation of transformation products contributing to more than 10 % of the applied amount of parent compound was not expected, because the mass balances did not show any trend to decline after equilibrium was reached. This means that the substances have primary degradation times longer than 100 days that are most likely exceeding the trigger value for very persistent substances.

**Table 5:** Results of the study by Wick et al. showing the high DT50 values in a non-GLP study comparable to OECD 308

	UV-327	UV-328	UV-350
Recovery at day 0 (%)	<70	<70	<70
Recovery at day 16^a^ (%)	129 ± 12	122 ± 15	93 ± 6
Recovery at day 100 (%)	136 ± 12	142 ± 28	99 ± 9
Relative amount sorbed after day 16^a^ (%)	>99.5	>99.5	>99.5
Estimated log *K*_OC_	>4.4	>4.4	>4.4
DT50 (water) (days)	<2	<2	<2
DT50 (sediment) (days)	>100	>100	>100
DT50 (total) (days)	>100	>100	>100

^a^Sorption equilibrium reached

#### Simulation field study

Recently, Lai et al. [42] examined the dissipation behaviour of several phenolic benzotriazoles including UV-327 and UV-328. In the study, dewatered sludge containing phenolic benzotriazoles was applied onto agricultural land. In a first experiment, this was done only once while in a second experiment application was repeated for 4 years. On the fields wheat and maize were cultivated. After treatment, soil sampling was performed monthly in the warm period of the following year. Based on the concentrations of phenolic benzotriazoles found after extraction, the authors studied the disappearance by applying dynamic curve fitting. The resulting half-lives are shown in Table 6. The results have to be considered as best case estimations of DT50 for several reasons: they only reflect the warmer period of the year; 3 years passed between (first) application and measurements, therefore potentially allowing microorganisms to adapt; only dissipation was monitored including all possibilities of substance losses, and finally NERs were not considered at all. Nevertheless, the DT50 values calculated are between 151 and 218 days.

**Table 6:** Overview of the reported DT_50_ values (dissipation in the field) by Lai et al. [42]

Substance	UV-327		UV-328	
	T1	T2	T1	T2
DT_50_ (days)	151	192	179	218
Error (days)	19	28	27	42

#### Simulation laboratory study according to OECD 308 using the substance EC 407-000-3 (P)

**Fig. 2:**
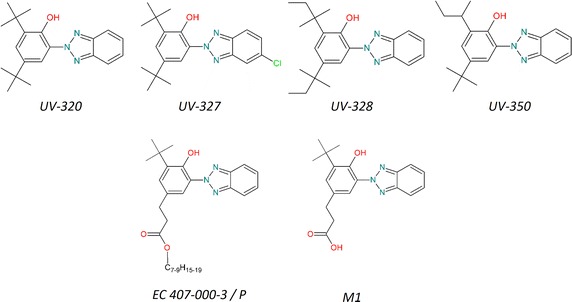
Structures of the four assessed phenolic benzotriazoles as well as those from the simulation study

Additionally to the studies described so far, information from a water–sediment study according to OECD 308 on dissipation and degradation of a similar substance (EC 407-000-3, for sake of brevity further on abbreviated as P) is available. This substance is defined as the reaction mass of branched and linear C7-C9 alkyl 3-[3-(2-H-benzotriazole-2-yl)-5-(1,1-dimethyl)-4-hydroxyphenyl]propionates. The study on this substance is used for read-across on the persistence of the four phenolic benzotriazoles due to structural homologies of its main metabolite M1 with the substances in question (see Fig. 2).

According to REACH Annex XI 1.5 (grouping of substances and read-across approach), the aim of a read-across is to avoid testing of every substance for every endpoint using data known for one substance for other, similar substances. Substance similarity may be based on three criteria:

1. a common functional group,
2. common precursors and/or the likelihood of common breakdown products via physical and biological processes, which result in structurally similar chemicals, or
3. a constant pattern in the changing of the potency of the properties.

All three criteria are met in this example. The structural similarity of P with the phenolic benzotriazoles under investigation is shown in Table 6 and Fig. 2. P consists of different 4,6-substituted phenolic benzotriazoles. Both substitution groups are alkyl chains. Position six is substituted with a *tert*-butyl group which is also present in UV-320 and UV-327. In UV-328, there is a *tert*-pentyl group, the next higher homologue of a *tert*-butyl group in position six. In case of UV-350, a *sec*-butyl group is in position six, which is a structural isomer of a *tert*-butyl group. Position four of the substances UV-320, UV-327 and UV-350 is again substituted with a *tert*-butyl group, while it is substituted with a *tert*-pentyl group in case of UV-328. P is substituted in position four of the phenolic ring with propionic esters of different lengths and branching patterns. The difference between UV-320 and UV-327 is a chlorine atom on the benzotriazole moiety. In summary, the four substances are structurally very similar to the reaction mass P.

Not only the substances themselves are similar, but according to a simulation with the EAWAG-BBD Pathway Prediction System (PPS) [43] also the breakdown products are similar. The PPS is the most comprehensive software to simulate degradation pathways. It is a rule-based system currently encompassing 249 microbial biotransformation rules based on over 1350 microbial catabolic reactions and about 200 biodegradation pathways. The system compares the organic functional groups of the molecules entered with its set of rules and shows all possible degradation steps. The reaction steps are colour coded according to the likelihood that the respective reaction is catalysed by certain bacteria in water, soil or sediment. An overview of the system can be found in two publications by Ellis et al. [44] and Gao et al. [45]. It is not possible to predict rate constants with this system. Also, there is no defined applicability domain for this rule-based system. The rules of the PPS were not explicitly derived for cleavage of phenolic rings bound to benzotriazole, and therefore it is uncertain if the mechanism proposed by is the most relevant one in the environment. However, as it consists only of common degradation reactions, they are nevertheless plausible predictions.

**Fig. 3:**
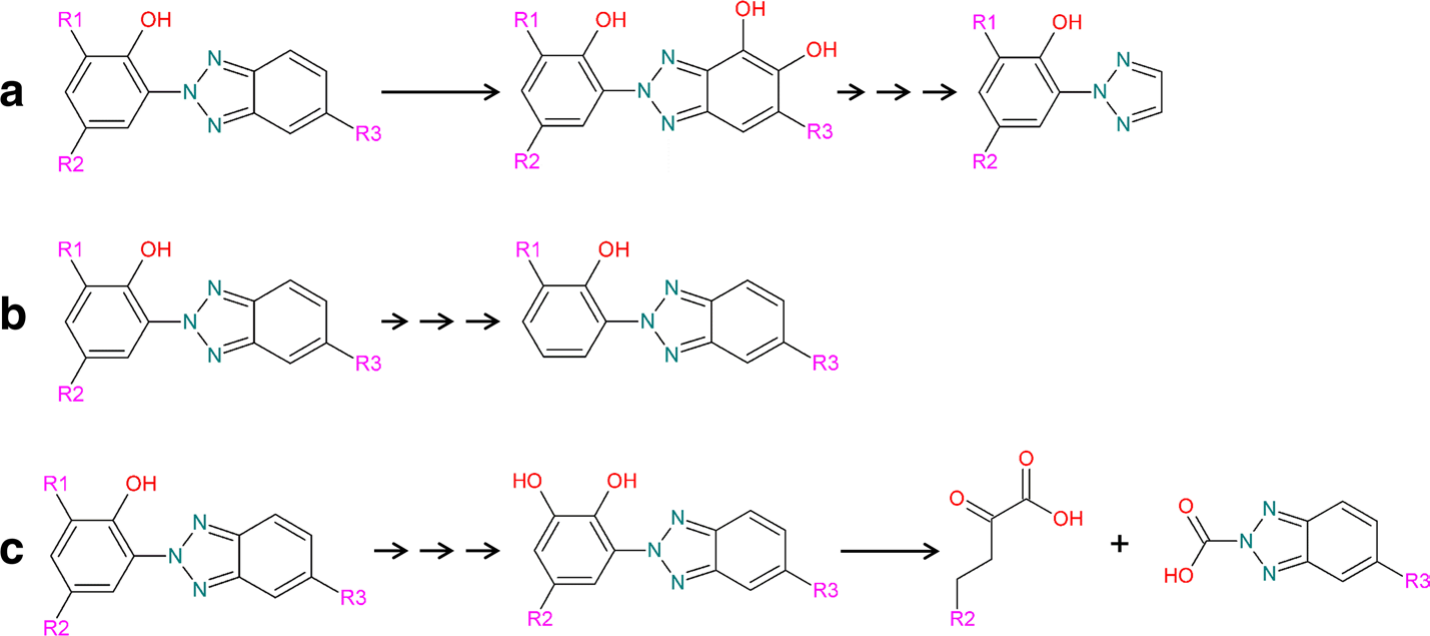
Simulated simplified mechanisms for the degradation of the phenolic benzotriazoles. **a** Degradation of the benzotriazole moiety, **b** degradation of side chain R2 and **c** degradation of side chain R1 leading to the ring cleavage of the phenolic ring R1, R2: alkyl; R3: H or Cl. Side reactions are for the sake of simplicity not considered here. Simulation was done using the EAWAG-BBD Pathway Prediction System [43]

The simulation shows that the stepwise degradation of the side chain in position four of P and its corresponding acid M1 (generated by fast enzymatic or abiotic ester hydrolysis) leads to a breakdown product that is also encountered when following the most probable transformation pathways of the phenolic benzotriazoles under consideration. Hence, the subsequent degradation steps will be identical. A simplified overview of the three idealized possible degradation pathways is shown in Fig. 3. Pathway (a) shows the first step of the degradation starting at the benzotriazole moiety that will stop when only the triazole group is remaining. Pathway (b) shows that the stepwise degradation of R2 will not lead to a breakdown of the phenolic moiety. Pathway (c) finally shows that the stepwise degradation of R1 can in the end lead to two vicinal hydroxyl groups resulting in the cleavage of the phenolic moiety, which is necessary for total mineralization. In the actual degradation of the phenolic benzotriazoles, all three possible degradation pathways will coexist and it is a question of the individual molecular structure of the metabolite which pathway is the kinetically most favourable.

Based on the chemical composition of the substitution groups of the four phenolic benzotriazoles and the metabolite M1, the order of the expected apparent degradation rates is estimated qualitatively as follows:

$$\begin{aligned} & {\text{DegT}}50({\text{M}}1) < {\text{DegT}}50({\text{UV-350}}) < {\text{DegT50}}({\text{UV-328}}) \\ & \quad \approx {\text{DegT50(UV-320)}} \approx {\text{DegT50}}({\text{UV-327}}). \\ \end{aligned}$$

The rationale for this qualitative order can be understood when considering the expected respective contributions to the ultimate degradation times of the individual side-chain fragments R1 and R2 of the phenolic moiety in the different molecules as shown in Table 7. The propionic acid should chemically be the easiest to degrade, followed by the *sec*-butyl group, the *tert*-butyl group and finally the *tert*-pentyl group. The last two will be harder to degrade due to the quaternary carbon atoms. This qualitative assessment is supported by the individual contributions of the fragments encoded in common QSAR models. Table 7 lists as example the contributions of the fragments in the BIOWIN3 model (ultimate biodegradability) of EPISUITE that support the assessment. Within BIOWIN3, the ultimate biodegradability is calculated by summation of the fragments and the higher the sum the higher the biodegradability. This means that fragments with lower value are actually harder to biodegrade and have accordingly a greater DT_50_.

**Table 7:** Fragments to be considered for qualitative assessment of degradation times

Substance	R1		R2		R3
	Name	Value of fragment within BIOWIN3	Name	Value of fragment within BIOWIN3	Name
M1	*Tert*-butyl	−0.34	*n*-Propionic acid	+0.20	Hydrogen
UV-350	*Sec*-butyl	−0.13	*Tert*-butyl	−0.34	Hydrogen
UV-328	*Tert*-pentyl	−0.37	*Tert*-pentyl	−0.37	Hydrogen
UV-320	*Tert*-butyl	−0.34	*Tert*-butyl	−0.34	Hydrogen
UV-327	*Tert*-butyl	−0.34	*Tert*-butyl	−0.34	Chlorine

Based on this expectation, the degradation rate determined for M1 should be higher than the those of the four phenolic benzotriazoles under consideration.

In summary, the overall REACH criteria for applying a read-across approach are met.

In the study using the substance P, an aerobic river system, an aerobic pond system and an anaerobic pond system were employed. The degradation of P and its metabolites was examined by radiolabeling P and measuring the radioactivity for 100 days. Only the corresponding carboxylic acid was identified as the first metabolite M1. Also eight further metabolites (M2–M9) were detected in lower concentrations but not identified. Thus, only apparent half-lives can be estimated. Furthermore, the evaluation of the test results is hampered by a high percentage of the radioactivity bound as NER. In case of the river system, after 100 days almost 40 % of the overall radioactivity in the sediment samples was detected in the NER fraction.

**Fig. 4:**
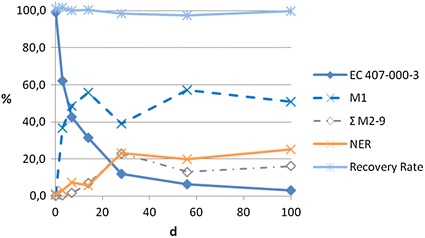
NER, parent, metabolites and total radioactivity in the whole system of the pond system under aerobic conditions

**Fig. 5:**
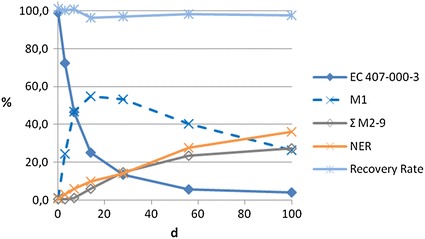
NER, parent, metabolites and total radioactivity in the whole system of the river system under anaerobic conditions

The results of the sediment fractions of the three test systems are shown in Figs. 4 and 5.

For modelling and fitting of the degradation kinetics, the software KinGUI Version 2.0 [46] was used. By following the stepwise procedure and kinetic models described in FOCUS Guidance on Estimating Persistence and Degradation Kinetics [47], the dissipation half-lives of M1 in water and sediment in both systems were estimated as well above 200 days each. The results are shown in Table 8. As this is a best case estimation for the substances actually in question, this means that their dissipation half-lives should be at least as long.

**Table 8:** Summary of dissipation half-lives of M1 for water and sediment under different test conditions (DT_50_ values for 20 °C)

Water/sediment study (OECD 308) for metabolite M1 of P	Water DT_50_^a^ (days)	Sediment DT_50_^a^ (days)
Aerobic conditions		
River system (low org. C)	3	32
Pond system (high org. C)	4	248
Anaerobic conditions		
Pond system (high org. C)	12	238

^a^The KinGUI-software does not compute the significance of the individual DT_50_-values. Given the few data points and their trend it is rather low, but for the sediment of the pond systems well above the relevant trigger value of 180 days

#### Case studies on degradation of phenolic benzotriazoles in the environment

**Fig. 6:**
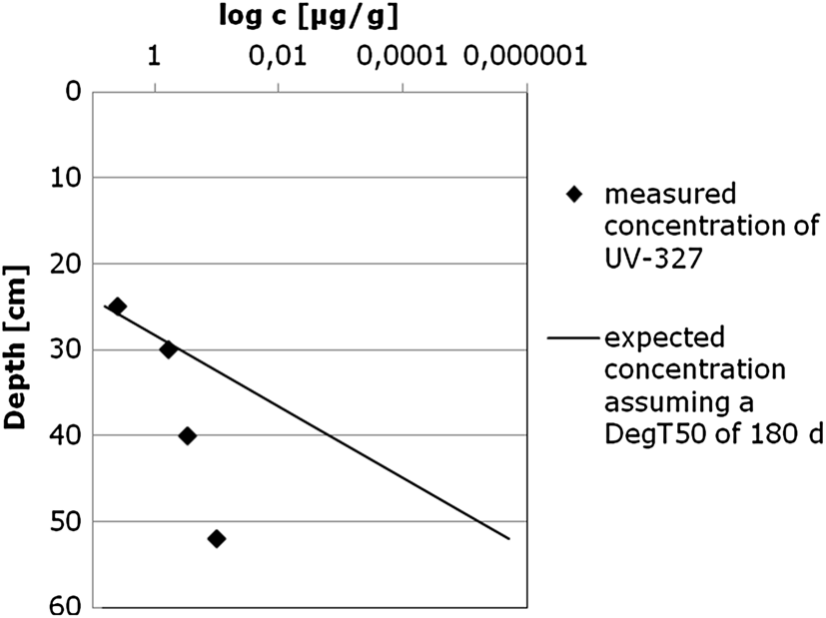
Graphical plot of the measured concentrations of UV-327 in different depths. Also included is a comparison with the concentrations that would be measured, if UV-327 had a DegT_50_ of 180 days. Note that the concentration scale is logarithmic

For UV-327 and UV-328, four studies are available on the distribution in sediments in a highly contaminated area (Narragansset Bay, RI, USA). These data deliver additional evidence on the slow degradation of all four phenolic benzotriazoles in sediments. UV-327 and UV-328 were historically produced in an industrial plant at the Pawtuxet River, which contributes to the Providence River and consequently the Narragansset Bay [34, 48–50]. UV-327 was produced between 1963 and 1972, while UV-328 was produced from 1970 to 1985 [34, 51, 52]. Two of the studies provide information about the sediment concentration during the production phase of UV-328: Jungclaus et al. [49] analysed the industrial WWTP effluent, the receiving waters and sediments from the chemicals manufacturing plant. Lopez-Avila and Hites [34] investigated the same sediments. Concentrations decreased both with depth in the sediment and with increasing distance from the discharge. The two other studies provide some evidence about the concentration of the compounds years after the production ceased: Reddy et al. [48] examined the free and bound fractions of different substituted benzotriazoles in sediment cores from the Pawtuxet River and Narragansett Bay. The river sediment core was collected in 1989, i.e. 4 years after production of UV-328 and 17 years after production of UV-327 was ceased. The bay core was collected in 1997, i.e. 12 years after production of UV-328 and 25 years after production of UV-327. Only UV-327 was detected. Taking into account the known sedimentation rate, it can be concluded that exposure was 7–10 years before the actual measurement. When we assume that exposure was constant during the years, the decrease in the UV-327 concentration should reflect the degradation rate of UV-327. As a very rough estimate, concentration decrease in depth can be compared to a decrease which would be expected assuming a DegT_50_ of 180 days (see Fig. 6, assumption: 2.5 cm depth reflects 1 year). Although the uncertainties of these estimations need to be taken into account, this supports the assumption of a very slow degradation of UV-327 under anaerobic conditions.

Hartmann et al. [51] took sediment cores in Narragansett Bay in 1997, i.e. 12 years after production of UV-328 and 25 years after production of UV-327. Taking into account the specific sedimentation rates, it is possible to identify the layer which probably represents exposure during active production of UV-327 and UV-328. This might be used to compare concentrations with the historical concentrations found in the other two studies conducted during production in order to get an idea about whether or not degradation occurred. The comparison is shown in Table 9. While the study interpretations are uncertain, they indicate that the degradation of UV-327 and UV-328 in the environment is very slow under anaerobic conditions. Even 25 years after production ceased, concentrations of phenolic benzotriazoles are of the same or only slightly lower concentration range. This provides further support for the hypothesis that degradation of UV-327 and UV-328 in sediments is slow and that the degradation half-lives are larger than 180 days.

**Table 9:** Comparison of measured concentrations of UV-328 in the Pawtuxet River and the Narragansett Bay during and after its production

Study	Detection limit (ppm)	Site	Year of collection	Sedimentation rate (cm)	Layer assumed to reflect production period (cm)	*c* at that layer (ppm)	*c* (historical, but probably not at the exact same spot) (ppm)
UV-327 (production period 1963–1972)							
[48]	0.02	Pawtuxet River	1989	2–3	34–69	0.1 (at 52 cm)	20–300 [49]
[51]	0.01	Quonset Point (Narragansett Bay)	1997	2	54–68	0.5 (at 50–60 cm)	0.5 [34]
[51]	0.01	Apponaug Cove (Narragansett Bay)	1997	0.5–0.85	14–29	0.07 (at 10–12 cm)	0.5 [34]
UV-328 (production period 1970–1985)							
[51]	0.01	Quonset Point	1997	2	24–54	0.04 (at 50–60 cm)	0.6 [34]
[51]	0.01	Apponaug Cove	1997	0.5–0.85	6–23	0.13 (at 10–12 cm)	0.6 [34]

### Summary of the WoE approach to assess the persistence of the four phenolic benzotriazoles

Combining the available information described above, the following is qualitatively concluded: UV-320, UV-327, UV-328 and UV-350 are phenolic benzotriazoles of very similar chemical structure. The simulations of degradation pathways show a common pathway in the stepwise degradation of the side chain in ortho position to the hydroxyl group. Therefore, the findings for the individual substances can be generalized and used for a read-across assessment. The substances for which tests on ready biodegradability are available show little or no degradation at all. Hence, these substances are potentially persistent by fulfilling the screening criteria according to REACH, Annex XIII. This is confirmed by QSAR calculations (with UV-350 being a borderline case). For UV-327, UV-328 and UV-350, it was shown in a laboratory simulation study that the substances bind to soil and sediment and that the DT50 values are longer than 100 days. The results of a simulation field study for UV-327 and UV-328 estimate the DT50 values for the substances to be 151–218 days under favourable conditions. For a closely related substance (P), aerobic and anaerobic simulation studies are available. The degradability of this substance and its main metabolite M1 can be used for a best case read-across meaning that UV-320, UV-327, UV-328 and UV-350 are expected to degrade at a lower rate and hence are assessed as being more persistent. Depending on the organic content of the system, the DT_50_ values (encompassing dissipation and degradation) in sediment under aerobic conditions will be approximately 248 days for M1 and under anaerobic conditions at least 238 days. Hence, M1 has to be considered as very persistent in the environment. This in turn means that the four phenolic benzotriazoles should be as well. A case study on sediments from a highly contaminated area in the US supports these findings. In this case, UV-327 and UV-328 were found in considerable amounts in deeper layers of the anaerobic sediment up to 25 years after the industrial releases of the substances ceased. The concentrations found can only be explained if the DegT_50_ of the substances is much longer than 180 days.

In summary, the available information from different sources implicates that the phenolic benzotriazoles under consideration are persistent in the environment. Moreover, the studies by Wick et al. (publication accepted) and Lai et al. [42], the simulation study on the similar substance P as well as the Pawtuxet River monitoring studies on UV-327 and UV-328 indicate that the degradation half-lives of the substances are longer than 180 days in freshwater sediments justifying an evaluation as very persistent in the REACH context.

### Assessment of the remaining uncertainty

According to our understanding, a convincing WoE assessment should encompass the assessment of the overall uncertainty. To do this again qualitatively, we will at first look again on the individual pieces of information and afterwards assess the overall uncertainty remaining when combining them.

#### Experimental tests and in silico simulations of ready biodegradability

The results of the tests on ready biodegradability are not suited for comparison with the numerical criteria of Annex XIII, because these screening tests only allow a decision whether a substance might be persistent or not T.

In the QSAR models used in our assessment, the triazole group is not represented and therefore not considered, but it is known that the benzotriazole substructure is not easily biodegraded [41] when adapted and active sludge is not used [52, 53], and therefore the results of the QSAR calculations on phenolic benzotriazoles will probably underestimate the persistence.

#### Simulation laboratory study similar to OECD 308

The main uncertainty with regard to the study of Wick et al. (publication accepted) is the limited duration of 100 days indicating only that DT_50_ values are clearly above 100 days. However, an extrapolation of half-lives and a comparison with the criterion of Annex XIII are not possible. Also while the study is similar to the OECD 308, it diverges in some points and has thus an overall lower reliability (reliability 2 according to the Klimisch Score [54]).

#### Simulation field study

The field study of Lai et al. [42] has some experimental shortcomings. The concentrations of the different benzotriazoles in the sludge and initial concentration values for the different field trials after the first and subsequent applications of the biosolids are missing. Furthermore, none of the metabolites were measured or determined. Finally, the limits of detection and quantification are quite high. To assess the overall method, the level of NERs would have had to be determined. A shortcoming for the use in our argumentation is that the study gives information on apparent disappearance (including degradation as well as dissipation) only and not degradation as required by Annex XIII.

#### Simulation test according to OECD 308 on P

The study on the substance P shows some general shortcomings associated with the evaluation of the test system for very lipophilic substances: the water solubility of P is very low. The substance strongly binds to organic carbon. This leads to an experimental complexity which renders the subsequent assessment difficult. The highest uncertainty in this particular experiment is associated with the very high fraction of NER. Since only the first metabolite of P (M1) was identified, no degradation half-lives can be calculated for complete mineralization and only estimations of apparent disappearance half-lives are possible.

Additional uncertainty arises from the evaluation from the two pond systems.

The DT_50_ result for the aerobic pond system is certainly influenced by the fact that the last two data points of the concentration of M1 in sediment seem to indicate that either a plateau is reached or a very slow decline is beginning. If the associated errors of the concentration values would be known or if there were more data points at the end of the experiment, it would be possible to perform a sensitivity analysis on the resulting DT_50_ values depending on these points. Given the available information, the derived numeric value should be considered with great caution as it might vary, but it is unknown by how much, since the test duration was considerably shorter than the estimated DT_50_ (100 vs. approximately 240 days).

In case of the anaerobic pond system, only a small part of the degradation curve of M1 is considered. Up to day 100, M1 is still formed and the maximum is not reached yet. Therefore, it is unknown how the actual disappearance curve looks like. If it follows the same trend as in the aerobic pond, the resulting DT_50_ value would be higher than the one calculated and maybe even higher than calculated for the aerobic pond (as from a biological and chemical point of view it should be).

Nevertheless, the overall DT_50_ values obtained with the aerobic and anaerobic pond test systems are very high and they can be considered as best case estimations. The degradation half-lives of the phenolic benzotriazoles are expected to be higher than the estimated disappearance half-lives of the proxy substance, but it is uncertain to which extent.

#### Case study of degradation of phenolic benzotriazoles in the environment

As our case study of degradation of phenolic benzotriazoles in the Pawtuxet River and the Narragansett Bay comprises four individual studies by different authors, drawing overall conclusions is associated with some uncertainty. The four studies had different purposes and used different methods, the sampling sites are different and the samples are sometimes not well described. As the number of sampling sites is limited, it is uncertain whether the findings can be generalized, as there might have been events that disturbed the sediment layers, e.g. storms, floods, bioturbation, etc. Finally, the sediment layer samples all seem to be anaerobic, while usually aerobic sediments are used for degradation assessment. Therefore, it is merely possible to state generally that the contaminant levels during production and 12–25 years after production are of the same level or only slightly different. Assuming that the sediment layers were not disturbed in these years, the degradation was very slow.

### Overall assessment of uncertainty

Each of the different information sources shows by itself limitations, deficiencies or uncertainties. Considering each information source on its own, it is impossible to conclude with ample confidence that phenolic benzotriazoles are very persistent in the environment according to the requirement defined by REACH. However, as all pieces of information used in this WoE approach are independent of each other, it is possible to combine the information pieces into a broader picture disregarding individual shortcomings. In this picture, the overall level of uncertainty becomes much lower, because all the information considered indicates a high persistence. The WoE approach is intended exactly for such cases.

## Conclusions

The weight-of-evidence approach presented here is useful to assess the persistence of certain 4,6-substituted phenolic benzotriazoles. The approach is following the criteria of Annex XIII of the European REACH Regulation, and the guidance documents established by the ECHA. The case study explores possibilities and the flexibility of the WoE approach in environmental assessment in general and under REACH in particular.

In this case, an assessment was possible although crucial standard information is not available for the substances under consideration. A variety of independent information sources were taken into account. Separately, these sources are insufficient to justify a clear conclusion. However, taking into account the information sources jointly, a comprehensive assessment is leading to the conclusion that the substances need to be assessed as very persistent.

Under the REACH framework, the WoE approach was used in the regulatory practice so far in only a few cases. For this case, several rounds of discussion within expert groups were necessary and the first attempt to identify these substances even failed as the experts were not able to decide on this new kind of approach. In the second attempt, the experts and decision makers followed our assessment. The overall decision-making process on this case has taken 4 years.

What is still missing in general is a better common understanding of what should make up a reliable and valid WoE assessment. With regard to the assessment of persistence, the case introduced here seems to represent a useful approach: every relevant piece of information is assessed by itself and conclusions are drawn and then combined into an overall purely qualitative assessment. The same was done for the uncertainties of the individual studies and the overall assessment. These kinds of WoE approaches could be used in REACH registration dossiers as well as in regulatory proposals.

As it is now, a framework for using the WoE approach needs to be developed further to agree on a common strategy among authorities and registrants under REACH. More experience is needed for its successful implementation and many open questions remain. The relevant ECHA guidance document (“How to report weight of evidence”) is focusing mainly on the technical issues of reporting a WoE approach in the registration dossier. Practical issues, case studies and standardized reporting formats need to be provided.

A general challenge when using the WoE approach is the need of expert judgment. Expert judgment is by definition a subjective matter based on personal experiences. However, a standard process like SVHC identification requires clear guidance and a common understanding leading efficiently to unanimous decisions. One way forward would be to introduce methods of quantification in WoE approaches. While this would lower the influence of subjectivity somewhat, it would also lower the flexibility and regulatory applicability of these kinds of assessment schemes. Thus, the advantages and disadvantages should be well reflected before codifying further guidelines.

Despite the remaining practical challenges with regard to implementing the WoE approach, the importance of WoE will increase in the future of REACH, and beyond. It can be a viable alternative to unnecessary or even unreliable testing for registrants and authorities. By assessing information already available, further testing might be avoided and the costs for registrants might be reduced. In addition, efficiency of the regulatory practice will increase. Especially, when assessing the phase-in substances of the third and last registration deadline in 2018 WoE approaches will need special consideration. As these substances each have an overall tonnage between 1 and 100 t/a, there are fewer data required for registration. It remains to be seen of which quality these data will be and whether they will be sufficient for an assessment. Using all available information in a WoE approach, these problems might partly be overcome.

## Methods

Study reports and publications were collected in order to assess the persistence of phenolic benzotriazoles in the environment. All studies were assigned a reliability score according to Klimisch et al. [54]. For assessing ready biodegradability of the substances, tests according to OECD test guidelines 301 B and C available for three substances were reviewed. QSAR simulations with BIOWIN models 2, 3 and 6 of the QSAR software package EPISUITE [40] were used according to REACH guidance R.11 [55]. The available experimental studies were evaluated according to guidance R.11 and the Technical Guidance Document on Risk Assessment [56]. KinGUI Version 2.0 [46] was used for modelling degradation and the BBD PPS [43] to model possible degradation pathways and metabolites.

For the WoE approach, the REACH guidance document “How to report weight of evidence”, published by the ECHA [57] was consulted. As described there, expert judgment is used to draw conclusions on the evidence found. All data relevant for the assessment were evaluated and qualitatively weighed. Afterwards, a chain of evidence was drawn in the narrative summary of the approach and the overall remaining uncertainty was assessed as well.

## Abbreviations

B: bioaccumulative
BOD: biological oxygen demand
CAS: chemical abstracts service
d: days
DegT50: time interval after which 50 % of a substance is degraded
DT50: time interval after which 50 % of a substance is degraded or disappeared otherwise from the test medium
DisT50: time interval after which 50 % of a substance disappeared from the test medium (no degradation)
Dw: dry weight
EC: European Community
ECHA: European Chemicals Agency
EU: European Union
G: grammes
h: hour
*K*_ow_: octanol/water partition coefficient (log value)
l: litres
lw: lipid weight
m^3^: cubed metres (volume)
M1: Metabolite of EC 407-000-3 after ester hydrolysis
MITI: Ministry of International Trade and Industry (Japan)
mg: milligrams
Mol: moles
µg: micrograms
n.d.: not detected
NER: non-extractable residues
NOEC: no-observed effect concentration
OECD: Organisation for Economic Co-operation and Development
P: persistent also short-hand notation for substance EC 407-000-3
PBT: persistent, bioaccumulative and toxic
POP: persistent organic pollutant
PPB: parts per billion
PPM: parts per million
PPS: pathway prediction system
QSAR: quantitative structure–activity relationship
REACH: Registration, Evaluation, Authorisation and restriction of Chemicals Regulation (EC 1907/2006)
SVHC: substances of very high concern
T: toxic (hazard classification)
US: United States of America
UV: ultraviolet
UV-320: 2-benzotriazol-2-yl-4,6-di-*tert*-butylphenol, CAS 3846-71-7
UV-327: 2,4-di-*tert*-butyl-6-(5-chlorobenzotriazol-2-yl)phenol, CAS 3864-99-1
UV-328: 2-(2H-benzotriazol-2-yl)-4,6-ditertpentylphenol, CAS 25973-55-1
UV-329: a phenolic benzotriazole UV stabilizer, CAS 3147-75-9
UV-350: 2-(2H-benzotriazol-2-yl)-4-(*tert*-butyl)-6-(*sec*-butyl)phenol, CAS 36437-37-3
vB: very bioaccumulative
vP: very persistent
vPvB: very persistent, very bioaccumulative
ww: wet weight
WWTP: waste water treatment plant
